# A longitudinal study on the impact of breastfeeding with or without formula milk on dental caries

**DOI:** 10.1038/s41598-024-60582-w

**Published:** 2024-05-06

**Authors:** Sirima Sritangsirikul, Kemporn Kitsahawong, Oranart Matangkasombut, Ana Lucia Seminario, Waranuch Pitiphat

**Affiliations:** 1 Faculty of Dentistry, PhD Program in Oral Sciences, Khon Kaen University, Muang District, Khon Kaen, 40002 Thailand; 2 Department of Pediatric Dentistry, Faculty of Dentistry, Chulalongkorn University, Henri Dunant, Bangkok, 10330 Thailand; 3 Division of Pediatric Dentistry, Department of Preventive Dentistry, Faculty of Dentistry, Khon Kaen University, Muang District, Khon Kaen, 40002 Thailand; 4 Department of Microbiology and Center of Excellence On Oral Microbiology and Immunology, Faculty of Dentistry, Chulalongkorn University, Henri Dunant, Bangkok, 10330 Thailand; 5 Research Laboratory of Biotechnology, Chulabhorn Research Institute, Laksi, Bangkok, 10210 Thailand; 6 Timothy A. DeRouen Center for Global Oral Health, School of Dentistry, University of Washington, Seattle, WA 98195 USA; 7 Division of Dental Public Health, Department of Preventive Dentistry, Faculty of Dentistry, Khon Kaen University, Muang District, Khon Kaen, 40002 Thailand

**Keywords:** Diseases, Risk factors

## Abstract

Concerns exist about prolonged breastfeeding increasing dental caries risk, but evidence is mixed. This 2-year cohort study followed 486 toddlers, to examine the association between breastfeeding duration and caries at age 3. Caregivers reported feeding practices and potential confounders every 6 months. “Full breastfeeding” was defined as feeding breastmilk without formula milk regardless of other foods/liquids, whereas “any breastfeeding” was feeding breastmilk with/without formula milk. A calibrated dentist performed dental examinations. We used multivariable log-binomial and negative binomial regressions to estimate relative risks (RRs) and 95% confidence intervals (CIs) for caries prevalence and severity, adjusted for confounders. At 3-year-old, 60.3% of children exhibited caries (mean decayed-and-filled-teeth, dft: 3.3). Notably, full breastfeeding for 6–17 months reduced caries prevalence (RR = 0.84, 95%CI 0.73–0.98 for 6–11 months; RR = 0.78, 95%CI 0.63–0.96 for 12–17 months). Conversely, any breastfeeding ≥ 18 months significantly increased caries risk (RR = 1.45, 95%CI 1.31–1.60). Full breastfeeding ≥ 6 months or any breastfeeding 6–17 months was associated with lower dft scores in children. Our findings suggest a complex relationship between breastfeeding duration and caries. Full breastfeeding for moderate durations (6–17 months) offers protective benefits, while any breastfeeding ≥ 18 months increases risk in this population.

## Introduction

Dental caries is one of the most common chronic diseases in children. Over half a billion children worldwide experience untreated caries in primary dentition^1^, which could significantly impact their growth, development, and quality of life^2^. Etiology of dental caries is complex and multifactorial, with many risk factors related to lifestyle and behaviors, especially diets^3^. Because milk is the main source of nutrition for infants, breastfeeding and formula feeding practices are likely contributing factors for caries development in children.

The unique properties of human breastmilk make it the best source of essential nutrients for infants. It is well-documented that breastfeeding provides a multitude of health benefits for mothers and children^4,5^. Importantly, breastfeeding also helps build a strong bond between mothers and children. Thus, breastfeeding is highly supported by leading organizations worldwide. The American Academy of Pediatrics (AAP) recommends that “infants should be exclusively breastfed for 6 months and continued breastfeeding along with the addition of appropriate complementary foods through the first year of life and beyond, for as long as mutually desired by mother and child”^5^. The World Health Organization (WHO) and United Nations Children’s Fund (UNICEF) recommend breastfeeding for ≥ 2 years^6^. However, despite its numerous advantages for children, the *Lancet* series on breastfeeding identified caries in primary teeth as the only negative health consequence of breastfeeding if it continues beyond the first year of life^4^.

The association between breastfeeding duration and caries has been examined in several studies; however, the results remain contradictory. While some studies reported that prolonged breastfeeding could be a risk factor for caries in primary dentition^7–13^, others showed that breastfeeding was either protective^14,15^ or had no effect on early childhood caries^16–18^. Two systematic reviews and meta-analyses suggested that breastfeeding may protect against caries but breastfeeding beyond 12 months is associated with increased caries risk^19,20^. A recent systematic review, considering evidence from two cohort studies, indicated that breastfeeding up to 24 months does not increase caries risk, but a longer duration does^21^. These heterogenous findings justify the need for additional research, particularly cohort studies with careful control of relevant confounders. This prospective study aimed to investigate the association between breastfeeding duration and caries experience at 3 years of age. We hypothesized that breastfeeding for ≥ 12 months is a risk factor for caries in the primary dentition.

## Methods

### Study design and participants

The study protocol was conducted in accordance with the Declaration of Helsinki and approved by the institutional review boards of Khon Kaen University (HE592266) and University of Washington (HSD52258). Parents and/or caregivers provided written informed consent on behalf of the children for participation in the study.

**Figure 1:**
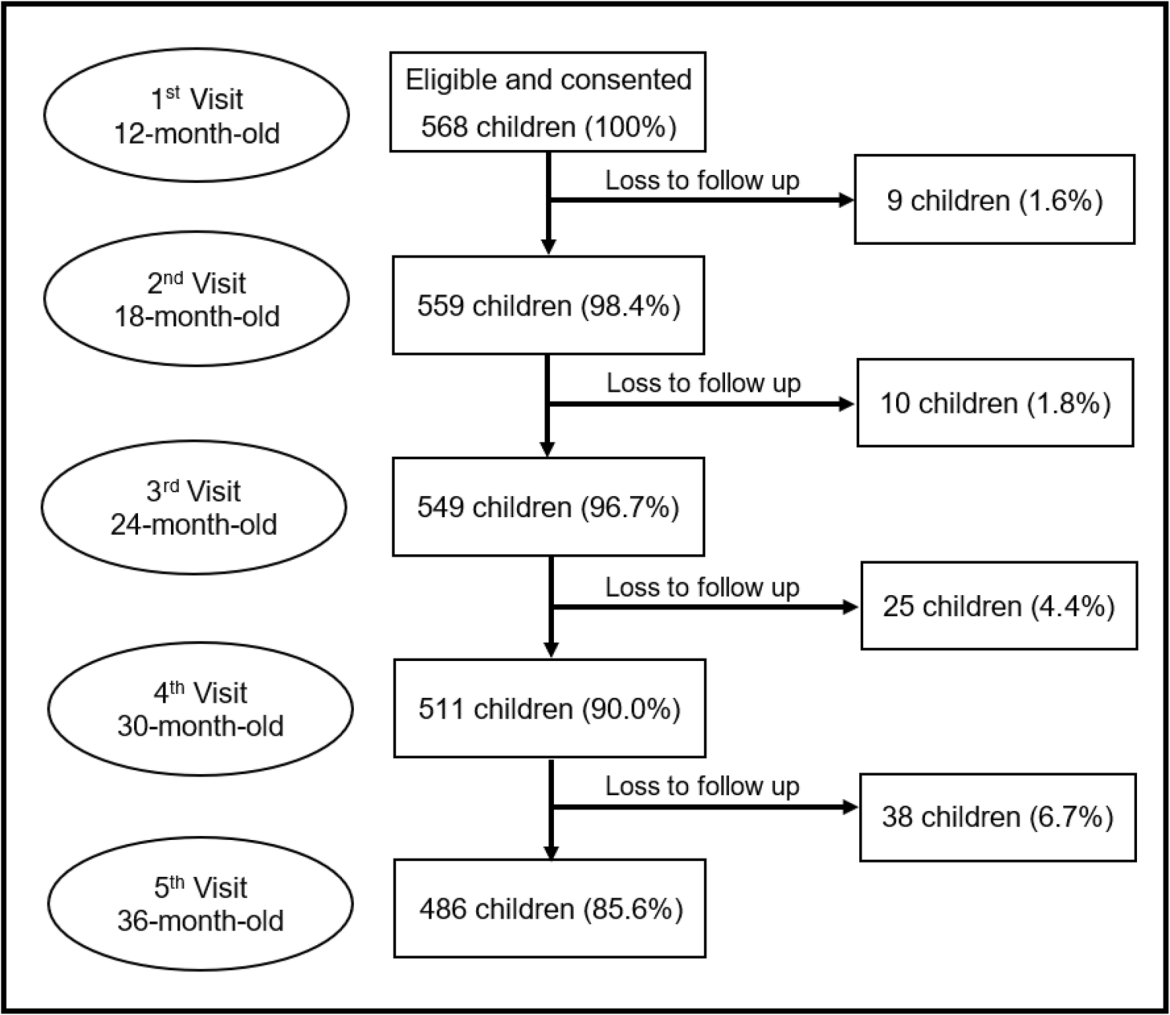
Flow chart of child participants through various stages of the study. All participants lost to follow-up were due to relocation to another district or province.

This prospective cohort study was conducted in Muang District of Khon Kaen, in the northeast part of Thailand. Participants included all healthy one-year-old children (± 3 months) who attended local public health facilities for routine immunization between January and April 2017. Eighteen public health facilities were randomly selected based on geographical locations to cover all areas of the district. Of the 596 invited, 568 children were recruited after excluding those whose mothers planned to relocate. These children were followed up every 6 months for 2 years. The present analysis was based on children who remained in the cohort at three years old (n = 486) (Fig. 1).

### Exposure and confounder assessment

Information on breastfeeding and other relevant factors was obtained through face-to-face interviews with mothers/caregivers by one interviewer using a structured questionnaire modified from our previous study^14^. Data on breastfeeding duration were collected at baseline and each follow-up visit through questions on breastmilk and other milk feeding habits, and age of weaning. This yielded a total of five surveys. Using the classification by Scott et al*.*^22^, breastfeeding was defined as: (1) full breastfeeding (providing an infant with breastmilk as the main source of nutrients, with or without water, other liquids, or foods, but not formula milk) and (2) any breastfeeding (providing an infant with breastmilk, with or without water, other liquids, foods, or formula milk). Additionally, children were classified according to their breastfeeding duration into four groups: < 6, 6–11, 12–17, and $\ge$ 18 months, following our previous work^14^. Full and any breastfeeding were classified separately.

Potential confounding factors were selected based on a review of the literature. Information collected at baseline include socio-demographic data of mothers/caregivers, frequency of teeth cleaning, fluoride toothpaste uses, and feeding habits (frequency of sleeping while milk feeding, frequency of water drinking after milk feeding, and number of meals per day). Information on sugar consumption between meals, including sugary drinks, sugary snacks, candies, jellies, desserts, and sweet fruits, was obtained at baseline and 3 years. Age of introduction to sugary foods was determined by interviewing mothers/caregivers at baseline and at each follow-up visit using the question, ‘Did your child consume any sweets between meals during the day or at night last week?’ Based on the responses collected across five surveys, we categorized the age of sugar introduction into three groups: < 12, 12–24, and > 24 months. The presence of visible plaque on buccal and lingual tooth surfaces was evaluated by a dentist at baseline and at 3 years, before caries assessment.

### Outcome measurements

Dental caries was assessed when the children reached three years old. A calibrated dentist performed full-mouth examinations using modified WHO diagnostic criteria^23^. The numbers and locations of tooth surfaces with cavitated and non-cavitated (white spot) lesions were recorded separately. A child was considered to have caries if any cavitated carious lesions were present in at least one tooth. Before the caries assessment, all tooth surfaces were carefully wiped with dry gauze to remove plaque. Examinations were conducted under artificial light, using a mouth mirror, while the children were in a supine position. The dentist and caregiver sat knee to knee. After the examination, the caregivers received a report indicating whether further treatment was needed. Caregivers whose children had painful dental problems were instructed to take their children to see a dentist at a nearby hospital.

### Statistical analysis

The baseline characteristics between children who completed the follow-up and those who dropped out were compared using Student’s t-test for continuous variables and Pearson’s Chi-square test for categorical variables. To compare participants’ characteristics across the categories of breastfeeding duration, Pearson’s Chi-square test or Fisher’s exact test was used for categorical variables, and one-way ANOVA or Kruskal–Wallis test for continuous variables. To determine the association between breastfeeding duration and caries prevalence, log-binomial regression with a generalized linear model was performed to estimate relative risks (RRs) with 95% confidence intervals (Cis). Variables with a *p* value < 0.10 in the bivariate analysis or identified as potential confounding factors were considered for inclusion in the multivariable analyses. The change-in-estimate method^24^ was employed to determine the inclusion of confounders. Confounders that changed the RR > 10% were included in the final model. Given that sugar consumption between meals was considered an important confounder, it was included in the final model, even though it changed the RR < 10%. To examine the association between breastfeeding duration and caries severity, the number of decayed and filled teeth (dft) was used as a count variable, and a negative binomial regression with a generalized linear model was employed to estimate RRs with 95% CIs. These models were fitted for overdispersion of caries incidence, assuming that the caries increment in this study had no excess zero. The analyses were performed using Stata version 10.1 (StataCorp, College Station, TX, USA). All statistical tests were two-sided, and a *p* value < 0.05 was considered as statistically significant.

The sample size was calculated with a ratio of 2 unexposed to 1 exposed, considering that 33.3% of Thai children continued breastfeeding after 12 months^25^. Based on a previous study^14^, caries prevalence among children aged 3–4 years who were breastfed for ≥ 12 months and < 12 months were 93% and 84.2%, respectively. A sample size of 477 was required to estimate the effect of breastfeeding for ≥ 12 months on dental caries, with 80% power and a 5% significance level.

## Results

The flow of participants through the various stages of the study is shown in Fig. 1. Of the 568 eligible children, 486 (85.6%) were present at all visits and included in the analysis. There was no significant difference in all characteristics between children who remained in the study and those who dropped out (Table 1). The children were evenly distributed by sex, with an average age of 12.8 (standard deviation, SD = 1.1) months at baseline and 35.6 (SD = 1.4) months at the caries assessment visit.

**Table 1:** Baseline characteristics of the participants included in the analysis and those who dropped out.

	Analyzed (N = 486)	Drop out (N = 82)	*p* value
**Child characteristics**			
Age in months, mean (SD)	12.8 (1.1)	12.6 (1.1)	0.30 ^†^
Sex			
Boy	50.2	51.2	0.87 ^‡^
Girl	49.8	48.8	
**Main caregiver characteristics**			
Main caregiver			
Mother	49	51.2	0.71 ^‡^
Other caregiver	51	48.8	
Age			
≤ 35 years	42.8	50	0.22 ^‡^
> 35 years	57.2	50	
Education			
Primary school or lower	43.2	34.1	0.26 ^‡^
Secondary school	38.9	47.6	
College or University	17.9	18.3	
Occupation			
Farmer/agriculturist/laborer	17.3	18.3	0.74 ^‡^
Professional or office worker	20	23.2	
Homemaker/unemployed	62.7	58.5	
**Maternal characteristics**			
Age			
≤ 35 years	86.4	91.5	0.21 ^‡^
> 35 years	13.6	8.5	
Education			
Primary school or lower	5.2	4.9	0.99 ^‡^
Secondary school	61.7	62.2	
College or University	33.1	32.9	
Occupation			
Farmer/agriculturist/laborer	14.8	14.6	0.74 ^‡^
Professional or office worker	50.4	46.4	
Homemaker/unemployed	34.8	39	
Maternal status			
Married	83.7	90.2	0.13 ^‡^
Single/divorced/separated/widowed	16.3	9.8	
Yearly family income			
< 120,000 THB	27	25.6	0.06 ^‡^
120,001–240,000 THB	35	22	
240,001–360, 000 THB	18.3	24.4	
360,001–1,800,000 THB	19.7	28	
Duration of full breastfeeding			
< 6 months	45	56.1	0.22 ^‡^
6–11 months	34.2	30.5	
12–17 months	15.4	8.5	
≥ 18 months	5.4	4.9	
Duration of any breastfeeding			
< 6 months	42.2	52.4	0.12 ^‡^
6–11 months	26.5	26.8	
12–17 months	13.2	4.9	
≥ 18 months	18.1	15.9	

Data presented as %, unless otherwise stated.

N, sample size; SD, standard deviation; THB, Thai Baht (1 USD = 35.74 THB).

^†^Student’s t-test, ^‡^Pearson Chi-square test.

Regarding breastfeeding, none of the children were exclusively breastfed for at least six months. More than half of the children were on full breastfeeding (55%) or any breastfeeding (57.8%) for at least 6 months. While any breastfeeding was continued in 18.1% of children for at least 18 months, full breastfeeding was continued in only 5.4% of children in the same duration (Table 1).

Mothers were the main caregivers of half of the children. Those raised by mothers or whose caregivers were younger, unemployed, and had education at the secondary school level or higher, tended to have a longer period of full or any breastfeeding (Table 2). Approximately two-thirds of the children who were breastfed for ≥ 18 months reported sleeping during milk feeding more than three times a week. Children who were breastfed for a longer duration were more likely to have at least three meals per day and tended not to drink water after milk feeding.

**Table 2:** Baseline sociodemographic characteristics and potential confounders according to the duration of breastfeeding (n = 486).

	n	Duration of full breastfeeding in months (%)					Duration of any breastfeeding in months (%)				
		< 6 (n = 219)	6–11 (n = 166)	12–17 (n = 75)	≥ 18 (n = 26)	*p* value	< 6 (n = 205)	6–11 (n = 129)	12–17 (n = 64)	≥ 18 (n = 88)	*p* value
**Main caregiver characteristics**											
Main caregiver											
Mother	238	35.6	51.2	65.3	100	**< 0.001**^**†**^	34.6	45	64.1	77.3	**< 0.001**^**†**^
Other caregiver	248	64.4	48.8	34.7	0		65.4	55	35.9	22.7	
Age											
≤ 35 years	208	31.5	45.8	57.3	76.9	**< 0.001 **^**†**^	30.7	39.5	59.4	63.6	**< 0.001**^**†**^
> 35 years	278	68.5	54.2	42.7	23.1		69.3	60.5	40.6	36.4	
Education											
Primary school or lower	210	55.2	38.6	32	3.9	**< 0.001**^**†**^	55.6	43.4	31.3	22.7	**< 0.001**^**†**^
Secondary school/College or University	276	44.8	61.4	68	96.1		44.4	56.6	68.7	77.3	
Occupation											
Farmer/agriculturist/laborer	84	25.1	13.2	8	3.8	**0.003**^**†**^	26.3	10.9	12.5	9.1	**0.001**^**†**^
Professional or office worker	97	19.6	20.5	18.7	23.1		19.5	20.1	17.2	22.7	
Homemaker/unemployed	305	55.3	66.3	73.3	73.1		54.2	69	70.3	68.2	
**Maternal characteristics**											
Age											
≤ 35 years	420	85.8	89.2	85.3	76.9	0.36^†^	86.8	87.6	89.1	81.8	0.54^†^
> 35 years	66	14.2	10.8	14.7	23.1		13.2	12.4	10.9	18.2	
Education											
Primary school or lower	25	5.5	4.2	6.7	3.9	0.85^‡^	4.9	4.7	3.1	8	0.61^‡^
Secondary school or higher	461	94.5	95.8	93.3	96.1		95.1	95.3	96.9	92	
Occupation											
Farmer/agriculturist/laborer	72	18.7	13.9	9.3	3.8	**< 0.001**^**†**^	19.5	14	12.5	6.8	**< 0.001**^**†**^
Professional or office worker	245	57.5	50.6	38.7	23.1		57.1	54.2	39.1	37.5	
Homemaker/unemployed	169	23.8	35.5	52	73.1		23.4	31.8	48.4	55.7	
Yearly family income											
< 120,000 THB	131	26.5	22.9	33.3	38.5	0.42^†^	25.9	22.5	26.6	36.3	0.34^†^
120,001–240,000 THB	170	38.8	34.3	28	26.9		39.5	32.6	34.4	28.4	
240,001–360,000 THB	89	17.8	21.1	14.7	15.4		18	21.7	15.6	16	
360,001–1,800,000 THB	96	16.9	21.7	24	19.2		16.6	23.2	23.4	19.3	
** Child characteristics from birth to 36 months**											
Sex											
Boy	244	48.9	53.6	52	34.6	0.31^†^	50.7	47.3	54.7	50	0.81^†^
Girl	242	51.1	46.4	48	65.4		49.3	52.7	45.3	50	
Duration of full breastfeeding											
< 6 months	219						100	1.6	4.7	10.2	**< 0.001**^**†**^
6–11 months	166						0	98.4	21.9	28.4	
12–17 months	75						0	0	73.4	31.8	
≥ 18 months	26						0	0	0	29.6	
Start age of other-milk feeding											
< 6 months	203	92.7	0	0	0	**< 0.001**^**†**^	93.7	1.5	4.7	6.8	**< 0.001**^**†**^
6–11 months	177	7.3	97	0	0		6.3	97	21.9	28.4	
12–17 months	71	0	3	88	0		0	1.5	65.6	30.7	
≥ 18 months	35	0	0	12	100		0	0	7.8	34.1	
Age of introduction to sugar											
< 12 months	471	97.3	97.6	94.7	96.1	0.43 ^‡^	97.1	97.7	95.3	96.6	0.60 ^‡^
12–24 months	14	2.7	2.4	4	3.9		2.9	2.3	3.1	3.4	
> 24 months	1	0	0	1.3	0		0	0	1.6	0	
**Child characteristics at 12 months**											
Age in months, mean (SD)	486	12.7 (1.1)	12.8 (1.1)	12.7 (1.1)	13.2 (1.2)	0.18^§^	12.7 (1.1)	12.9 (1.1)	12.6 (1)	12.9 (1.1)	0.17^§^
Cleaning teeth											
Never	75	19.2	10.8	14.7	15.4	**0.03**^**†**^	18.5	10	15.6	15.9	0.07^†^
< 2 times/day	185	41.1	38	37.3	15.4		40.5	38.8	43.8	27.3	
≥ 2 times/day	226	39.7	51.2	48	69.2		41	51.2	40.6	56.8	
Fluoride toothpaste											
Yes	115	20.1	24.1	26.7	42.3	0.07^†^	20.5	24	23.4	30.7	0.31^†^
No	371	79.9	75.9	73.3	57.7		79.5	76	76.6	69.3	
Visible plaque											
< 15%	379	79.4	74.7	82.7	73.1	0.45^†^	78.5	73.6	78.1	82.9	0.44^†^
≥ 15%	107	20.6	25.3	17.3	26.9		21.5	26.4	21.9	17.1	
Sleeping while milk feeding											
Never	152	27.4	38.6	30.7	19.2	**0.045**^**†**^	27.3	43.4	32.8	21.6	**0.008**^**†**^
1–3 times/week	101	21	22.8	18.7	11.6		21.5	20.2	23.4	18.2	
> 3 times/week	233	51.6	38.6	50.6	69.2		51.2	36.4	43.8	60.2	
Drinking water after milk feeding											
Never	145	24.7	27.7	41.3	53.8	**0.001**^**†**^	22.4	28.7	35.9	44.3	**0.004**^**†**^
Sometimes	193	38.8	44	32	42.3		40	42.6	35.9	37.5	
Always	148	36.5	28.3	26.7	3.9		37.6	28.7	28.2	18.2	
Number of meals											
1–2	158	38.4	33.7	21.3	7.7	**0.002 **^**†**^	39	37.2	23.4	17	**0.001**^**†**^
≥ 3	328	61.6	66.3	78.7	92.3		61	62.8	76.6	83	
Sugar consumption between meals											
≤ 3 times/week	161	35.6	31.9	29.3	30.8	0.81 ^†^	35.1	33.3	28.1	31.8	0.84^†^
4–6 times/week	128	23.3	28.3	28	34.6		22.9	27.9	31.3	28.4	
≥ 7 times/week	197	41.1	39.8	42.7	34.6		42	38.8	40.6	39.8	
**Child characteristics at 36 months**											
Visible plaque											
< 15%	407	81.3	83.1	88	96.2	0.18^†^	82.9	85.3	82.8	84.1	0.95 ^†^
≥ 15%	79	18.7	16.9	12	3.8		17.1	14.7	17.2	15.9	
Sugar consumption between meals											
≤ 3 times/week	107	23.3	24.1	12	26.9	0.17^†^	23.4	24	18.8	18.2	0.90 ^†^
4–6 times/week	98	20.1	18.7	28	7.7		19.5	20.2	23.4	19.3	
≥ 7 times/week	281	56.6	57.2	60	65.4		57.1	57.8	57.8	62.5	

Data presented as % unless otherwise stated.

N, sample size; SD, standard deviation.

^†^Pearson Chi-square test, ^‡^Fisher’s exact test, ^§^One-way ANOVA.

Boldface indicates statistical significance.

Of the 486 children, 293 (60.3%) had carious lesions at three years old. The prevalence and severity of caries were highest in children who were breastfed for less than 6 months. An inverse relationship between dental caries and breastfeeding duration was observed for full breastfeeding, i.e., dental caries decreased with a longer period of full breastfeeding. In contrast, the distribution of caries prevalence and severity by duration of any breastfeeding was U-shaped, with the shortest and longest duration groups being the most affected (Table 3 and Table 4). Intra-rater reliability was measured by repeating the examination in 10% of the samples. Cohen’s Kappa was 0.85, which was well above the expected minimum of 0.80.

**Table 3:** Association between duration of breastfeeding and prevalence of dental caries at 3 years of age (n = 486).

Duration	Full breastfeeding				Any breastfeeding			
	n	Prevalence of caries (%)	Unadjusted RR (95% CI)	Adjusted RR^†^ (95% CI)	n	Prevalence of caries (%)	Unadjusted RR (95% CI)	Adjusted RR^‡^ (95% CI)
< 6 months	219	66.2	1.0	1.0	205	66.8	1.0	1.0
6–11 months	166	56.6	0.86 (0.73, 1.01)	**0.84 (0.73, 0.98)**	129	51.9	**0.78 (0.64, 0.94)**	0.93 (0.77, 1.12)
12–17 months	75	54.7	0.83 (0.66, 1.04)	**0.78 (0.63, 0.96)**	64	53.1	0.79 (0.62, 1.02)	1.1 (0.85, 1.43)
≥ 18 months	26	50	0.76 (0.51, 1.12)	0.72 (0.50, 1.03)	88	62.5	0.94 (0.77, 1.13)	**1.45 (1.31, 1.60)**
*p* value		0.10 ^§^				**0.03** ^§^		

N, sample size; RR, relative risk; CI, confidence interval.

^†^Adjusted for age and education of the main caregiver, frequency of drinking water after milk feeding at baseline, visible plaque at baseline, and sugar consumption between meals at 36 months.

^‡^Adjusted for education of main caregiver, age when other-milk feeding was started, frequency of sleeping while milk feeding at baseline, and sugar consumption between meals at 36 months.

^§^Pearson Chi-square test.

Boldface indicates statistical significance.

**Table 4:** Association between duration of breastfeeding and dft score at 3 years of age (n = 486).

Duration	Full breastfeeding				Any breastfeeding			
	n	Mean dft (SD) median (min–max)	Unadjusted RR (95% CI)	Adjusted RR^†^ (95% CI)	n	Mean dft (SD) median (min–max)	Unadjusted (95% CI)	Adjusted RR^‡^ (95% CI)
< 6 months	219	3.9 (4.4)	1.0	1.0	205	3.9 (4.3)	1.0	1.0
		3 (0–20)				3 (0–20)		
6–11 months	166	3.0 (4.1)	**0.77 (0.61, 0.97)**	0.88 (0.69, 1.11)	129	2.8 (4.1)	**0.72 (0.56, 0.93)**	**0.76 (0.59, 0.99)**
		2 (0–20)				1 (0–20)		
12–17 months	75	2.7 (3.5)	**0.69 (0.51, 0.93)**	0.74 (0.54, 1.01)	64	2.8 (3.5)	**0.72 (0.52, 0.99)**	**0.7 (0.50, 0.98)**
		1 (0–13)				1.5 (0–13)		
≥ 18 months	26	2.1 (2.8)	**0.53 (0.33, 0.87)**	0.63 (0.38, 1.06)	88	3.2 (3.9)	0.84 (0.63, 1.12)	0.96 (0.72, 1.30)
		0.5 (0–11)				2 (0–14)		
*p* value		**0.03 **^§^				**0.02 **^§^		

N, sample size; dft, decayed and filled teeth; SD, standard deviation; RR, relative risk; CI, confidence interval.

^†^Adjusted for education of the main caregiver, frequency of sleeping while milk feeding at baseline, frequency of cleaning teeth at baseline, and sugar consumption between meals at 36 months.

^‡^Adjusted for education of main caregiver, frequency of cleaning teeth at baseline, and sugar consumption between meals at 36 months.

^§^Kruskal Wallis test.

Boldface indicates statistical significance.

Table 3 shows the association between breastfeeding duration and prevalence of dental caries. In the adjusted analysis, children who continued full breastfeeding for 6–17 months had a significantly lower risk of caries than those who received full breastfeeding for < 6 months (adjusted RR 0.84; 95% CI 0.73, 0.98 for 6–11 months and adjusted RR 0.78; 95% CI 0.63, 0.96 for 12–17 months). In contrast, any breastfeeding for ≥ 18 months was significantly associated with a 1.45 times increased risk of caries compared to any breastfeeding for < 6 months (adjusted RR 1.45; 95% CI 1.31, 1.60).

Regression analyses of the association between breastfeeding duration and caries severity are shown in Table 4. Compared with children who were fully breastfed for < 6 months, those who had a longer period of full breastfeeding had fewer teeth affected by caries. However, these differences were attenuated and became non-significant when adjusted for confounding factors. We observed a significantly lower dft score in children who continued any breastfeeding for 6–17 months compared to those who were breastfed for < 6 months (adjusted RR 0.76; 95% CI 0.59, 0.99 for 6–11 months and adjusted RR 0.70; 95% CI 0.50, 0.98 for 12–17 months).

**Figure 2:**
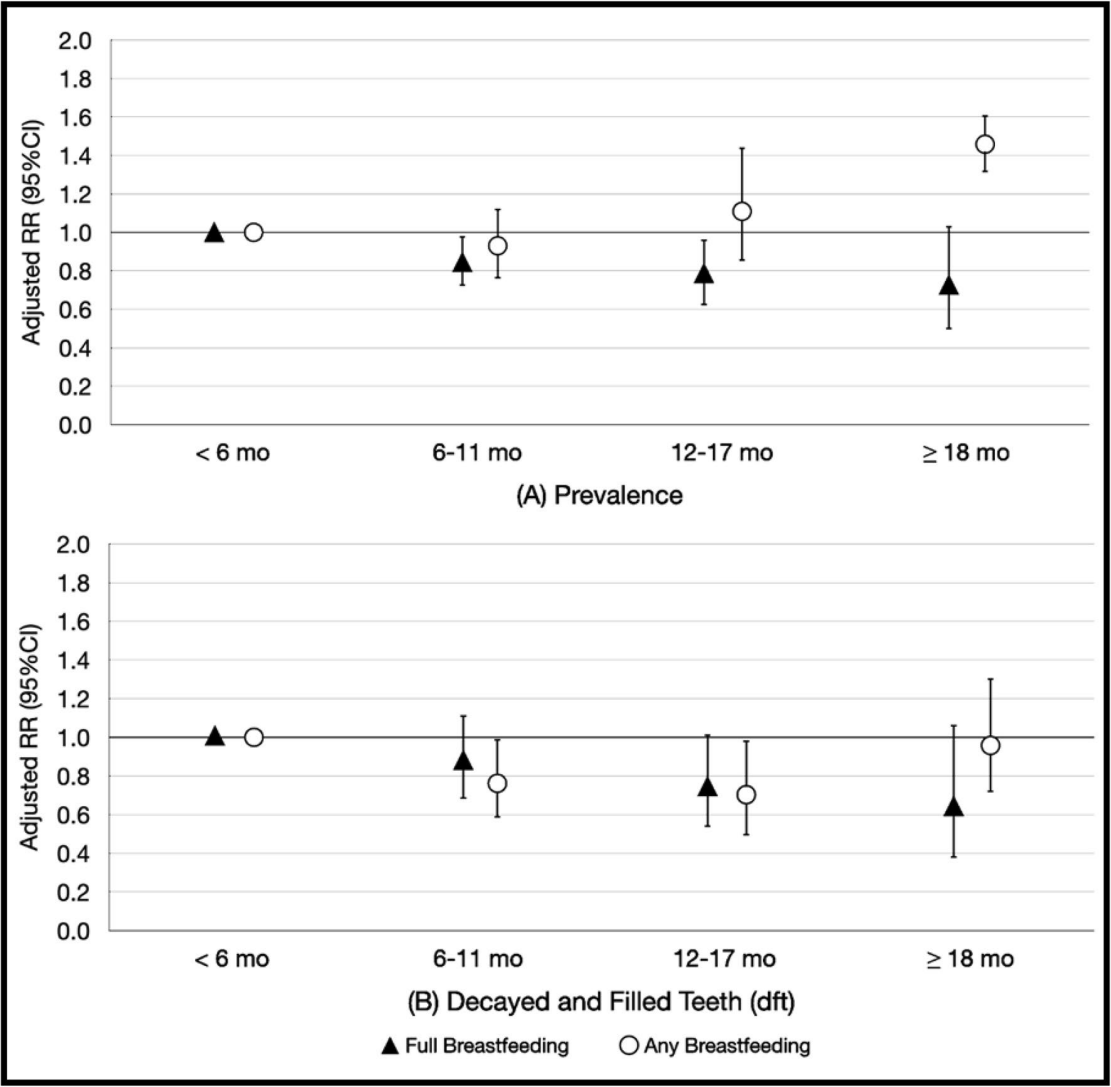
Adjusted relative risks of dental caries (**A**) prevalence and (**B**) decayed and filled teeth (dft) at 3 years of age by 4 breastfeeding groups.

Figure 2 illustrates the differences in the pattern of association between breastfeeding duration and caries experience according to full and any breastfeeding. Full breastfeeding duration was inversely associated with caries prevalence and dft in a linear fashion. In contrast, the association between any breastfeeding and dental caries was non-linear for both caries prevalence and dft.

## Discussion

This prospective study, conducted in a non-fluoridated urban area in Thailand, examined the relationship between the duration of full breastfeeding, any breastfeeding and dental caries, separately. After adjusting for important confounders, we found that full breastfeeding for 6–17 months decreased the risk of dental caries in 3-year-old children compared with full breastfeeding for < 6 months. Similar trend was also observed for full breastfeeding for ≥ 18 months, albeit not statistically significant, due to limited sample size in this group. In contrast, caries prevalence was increased when any breastfeeding was continued for ≥ 18 months, compared to any breastfeeding for < 6 months. These findings suggest that caries risk associated with prolonged breastfeeding is not a result of breastfeeding per se but is likely due to the addition of formula milk.

Only a few studies have examined breastfeeding type in relation to caries. A study using the US nationally representative data of children aged 2–5 years did not find an association between dental caries and duration of breastfeeding, whether it was exclusive, full or overall^18^. The limitation of cross-sectional data was addressed with a potential for recall bias in the estimation of breastfeeding duration and introduction of other foods. The present findings confirmed the results of our previous birth cohort study in a rural/semi-urban district of Khon Kaen, which reported that full breastfeeding beyond 6 months was caries-protective^14^. The tendency that prolonged duration of any breastfeeding might increase the risk of caries was also observed, although the results were not statistically significant.

Interestingly, our two cohort studies in non-fluoridated areas of Thailand found a non-linear association between the duration of any breastfeeding and caries experience, with < 6 months and ≥ 18 months groups being the most affected. These findings are consistent with a population-based cross-sectional survey in Australia that also observed a U-shaped association between breastfeeding duration and caries among children with no exposure to water fluoridation^7^. Non-linear J-shaped^10^ and a trend of U-shaped associations^26^ between breastfeeding duration and caries were also observed among young Japanese children in two non-fluoridated areas. These findings highlight the need to examine breastfeeding in intervals separating early (e.g., < 6 months) and late duration (≥ 18 months), rather than using specific binary cut-points^27^.

Our findings on any breastfeeding were in line with the results of previous systematic reviews. Tham and colleagues^20^ suggested a protective effect of breastfeeding for up to 12 months against caries but identified a higher risk of dental caries in children breastfed beyond 12 months. Another meta-analysis by Cui and colleagues^19^ reported that breastfeeding may protect against caries compared with never breastfeeding, while breastfeeding for ≥ 12 months was associated with an increased risk of dental caries compared to breastfeeding for < 12 months. Moynihan and colleagues also concluded that breastfeeding for ≤ 24 months does not increase caries risk, but a longer duration might elevate the risk^21^. A recent birth cohort study in the Netherlands confirmed the association between prolonged breastfeeding for > 12 months and an increased risk of childhood caries^13^. Nonetheless, another birth cohort study of Australian preschoolers (n = 965) did not observe an association between breastfeeding beyond one year and dental caries at 2–3 years of age. The authors addressed a limitation of the low prevalence of both dental caries and long duration of breastfeeding in this population, which could introduce a rare events bias^16^. A longitudinal study of Scottish children also failed to show the association of breastfeeding and dental caries at 1, 2, 3 and 4 years of age^17^. However, the study used 6 months as a cut-point for breastfeeding duration, which was not sensitive enough to detect the plausible effect of prolonged breastfeeding on caries^27^.

Mechanistically, there are several possible explanations for why prolonged full breastfeeding could decrease caries risk, while any breastfeeding beyond 18 months could increase the risk. The caries-protective effects may be derived from several glycoproteins in human breastmilk that could block the adhesion of *Streptococcus mutans*, a major cariogenic bacterial species, on tooth surfaces^28^. Moreover, the buffering capacity of breastmilk could help limit caries formation^29^ and allow remineralization of non-cavitated lesions. However, human breastmilk contains high levels of lactose that might cause a pH drop below the critical level for enamel dissolution^30^. The addition of other carbohydrate sources, such as formula milk, may lead to a pH drop that exceeds the buffering capacity and enhances caries development^29^. As the levels of caries-protective elements in breastmilk tend to decline beyond 12 months^31^, the balance may be easier to be tipped with additional formula milk in prolonged duration of any breastfeeding over 18 months.

In a cross-sectional study involving 1-to 3-year-old children across 10 Latin American countries, Feldens and colleagues^32^ found a relationship between breastfeeding duration and the age at which sugary products are introduced. The findings revealed that children who were breastfed for a shorter duration tended to be introduced to sugary products at an earlier age. However, in the current study, such a trend was not observed for either full or any breastfeeding (Table 2). This discrepancy might be explained by the high prevalence (97%) of children in our study receiving sugary foods before 12 months old. Other studies in Brazil found associations between early introduction to sugar^33^, increased exposure to cariogenic items before 12 months of age^34^, and the total daily feeding frequency at 12 months of age (including high-frequency breastfeeding and bottle-use)^35^ with dental caries development. Therefore, it is crucial to take into account formula milk and other carbohydrates in the diets of children when studying the association between breastfeeding and dental caries.

A major strength of our study is its longitudinal cohort design. We collected breastfeeding information prospectively at multiple time points to ensure accurate breastfeeding status and minimize recall bias from caregiver reports. We obtained data from primary caregivers, who were closely involved in looking after the children, ensuring that the data closely reflected the actual information. Caries examinations were performed by a single dentist who was unaware of breastfeeding status and had excellent intra-examiner reliability. The retention rate after two years of follow-up was high (85.6%), and the characteristics of the participants who remained and those who were lost to follow-up were similar (Table 1), suggesting that selection bias was unlikely. Notably, we tried to minimize confounding by collecting rich information on potential confounders identified in the literature to control for in statistical analysis. Furthermore, we examined full and any breastfeeding separately and classified the duration into four categories. This approach enabled us to elucidate the complex associations between breastfeeding and caries for each duration.

This study had some limitations. Information on feeding practices was collected through interviews. There is a potential for recall bias and social desirability bias which may lead to exposure misclassification, even though we gathered breastfeeding information prospectively at several time points to minimize bias. Although we adjusted for important confounding factors, there could still be residual confounding from unmeasured factors, such as frequency of sleeping during feeding and age at introduction of complementary foods. Finally, the generalizability of our results may be limited to the population with similar patterns of breastfeeding and ECC rates. However, the biological mechanisms linking breastfeeding duration to caries should be the same across populations.

In conclusion, our findings support the value of continued breastfeeding throughout the first year of life and beyond, as recommended by leading organizations. A longer duration of full breastfeeding can protect against early childhood caries. However, any breastfeeding (with or without formula milk) for ≥ 18 months increases the caries prevalence. Therefore, breastfeeding practices should be strongly encouraged, along with urging caregivers to provide proper oral hygiene and dietary practices for children. Further prospective cohort studies with detailed categorization of breastfeeding type (i.e., full and any breastfeeding) and duration are needed to strengthen this evidence.

## Data Availability

The data sets generated during and/or analyzed during the current study are available from the corresponding author on reasonable request.
